# Draft Genome Sequence of *Alcaligenes faecalis* Strain IITR89, an Indole-Oxidizing Bacterium

**DOI:** 10.1128/genomeA.00067-16

**Published:** 2016-03-03

**Authors:** Raj Kumar Regar, Vivek Kumar Gaur, Gayatri Mishra, Sudhir Jadhao, Mohan Kamthan, Natesan Manickam

**Affiliations:** a Environmental Biotechnology Laboratory, Environmental Toxicology Group, CSIR-Indian Institute of Toxicology Research (IITR), Vishvigyan Bhawan, Lucknow, India; b Bionivid Technology Pvt. Ltd., Bengaluru, Karnataka, India

## Abstract

We report the draft genome sequence of *Alcaligenes faecalis* strain IITR89, a bacterium able to form indigo by utilizing indole as the sole carbon source. The *Alcaligenes* species is increasingly reported for biodegradation of diverse toxicants and thus complete sequencing may provide insight into biodegradation capabilities and other phenotypes.

## GENOME ANNOUNCEMENT

*Alcaligenes faecalis* subspecies *phenolicus* strain IITR89 was isolated from the River Cauvery in Erode, India. The whole-genome sequence of strain IITR89 was obtained by an Illumina platform using NextSeq Illumina paired-end technology and produced a total of 34,630,080 bp paired-end reads of 151 bp. Next-generation sequencing quality-control (NGS QC) toolkit v2.3 (1) was employed to filter the data for high quality for genome assembly. A total of 31,151,224 high-quality filtered reads were used for assembly with Velvet v1.2.08 (2) (at a k-mer length of 69). Based on paired-end directional information, the genome was assembled into 28 scaffolds, with *N*_50_ length 535,462 bp and average scaffold length 134,770.18 bp using SSPACE v3.0 scaffolder (3). The obtained draft genome was assembled resulting in a total genome size of ~3.77 Mb with G+C content of 57.59 %. The genome draft of strain IITR89 consists of a pseudo chromosome and unaligned scaffolds. The final genome draft consists of 28 scaffolds with an average length of 134,770.18 bp (~134.8 Kb) and *N*_50_ size of 535,462 bp, constituting 3,774,065 bp of the genome.

The draft genome was annotated using Rapid Annotations using Subsystems Technology (RAST) Prodigal v2.6.2 (4), RAST v2.0 (5), and RNAmmer 1.2 servers (6). All three types of rRNAs and possible tRNAs were identified, indicating a high degree of completeness in the genome assembly. A total of 3,538 genes, 3,549 protein coding regions (CDSs), and 3 rRNAs (16s rRNA, 28s rRNA, and 5 s rRNA) were predicted from Prodigal. ARAGORN v1.2.36 (7) predicted 51 tRNAs. No plasmid was identified when it was analyzed using Webcutter (V 2.0) and PlasmidFinder (V 1.3) (8) and 1 prophage was identified using the PHAST server (9). The taxonomy identification method was performed using EzTaxon (10) and MEGA6, from which it was found that strain IITR89 is the putative species (as per sequence homology) and *Alcaligenes faecalis* subsp. *phenolicus* exhibits closest homology to *Alcaligenes faecalis* subsp. *faecalis*.

RAST annotation shows that strain IITR89 contains 3,046 characterized proteins, 538 hypothetical/putative proteins, and 1,039 proteins with pathway annotation. In the RAST annotation, we found genes coding for resistance to antibiotics and toxic compounds like arsenical pump-driving ATPase (EC 3.6.3.16), resistance to fluoroquinolones, i.e., DNA gyrase subunit B (EC 5.99.1.3), choloylglycine hydrolase (EC 3.5.1.24), bacteriocins, ribosomally synthesized antibacterial peptides like tRNA pseudouridine synthase A (EC 4.2.1.70), etc. Genes responsible for motility and chemotaxis, i.e., flagellar protein FlgJ (peptidoglycan hydrolase) and genes for oxidative stress hydroxyacylglutathione hydrolase (EC 3.1.2.6) are present. The strain also contains genes related to indole degradation like indole-3-glycerol phosphate synthase (EC 4.1.1.48) along with other dioxygenases and hydrocarbon degradation genes like catechol 1,2-dioxygenase (EC 1.13.11.1) and 3-carboxyethylcatechol 2,3-dioxygenase (EC 1.13.11.16), alkanesulfonate monooxygenase (EC 1.14.14.5), the 4-carboxymuconolactone decarboxylase domain/alkylhydroperoxidase AhpD family core domain protein, 3-oxoadipate CoA-transferase subunit B (EC 2.8.3.6), and 3-oxoadipate CoA-transferase subunit A (EC 2.8.3.6).

### Nucleotide sequence accession numbers.

This *Alcaligenes faecalis* strain IITR89 genome sequence has been deposited at EMBL/DDBJ/GenBank under accession number LQAS00000000. The version described in this paper is the first version, LQAS01000000.
